# ABA-Induced Transcriptomic Dynamics in *Arabidopsis thaliana* Anthers: Insights into Pollen Development and Fertility

**DOI:** 10.3390/plants15060894

**Published:** 2026-03-13

**Authors:** Lu Liu, Huiting Huang, Dexi Shi, Shuo Wang, Ziyi Lin, Fengming Huang, Li Huang, Sue Lin

**Affiliations:** 1 College of Life and Environmental Science, Wenzhou University, Wenzhou 325035, China; 23451039023@stu.wzu.edu.cn (L.L.); 355600230016@email.ncu.edu.cn (H.H.); 25351043004@stu.wzu.edu.cn (D.S.); 23451039039@stu.wzu.edu.cn (S.W.); 24451041019@stu.wzu.edu.cn (Z.L.); 24451041012@stu.wzu.edu.cn (F.H.); 2 Laboratory of Cell & Molecular Biology, Institute of Vegetable Science, Zhejiang University, Hangzhou 310058, China

**Keywords:** *Arabidopsis thaliana*, pollen development, abscisic acid (ABA), transcriptome sequencing, lncRNA

## Abstract

Pollen development is a complex process that is highly sensitive to environmental stresses. Abscisic acid (ABA), a key hormone mediating plant growth and stress responses, has been implicated in the regulation of sexual reproduction, especially pollen development, yet its precise regulatory role remains unclear. This study investigated the effects of exogenous ABA on *Arabidopsis thaliana* pollen development and function through integrated phenotypic, cytological, and transcriptomic approaches. ABA treatment specifically impaired pollen function by reducing germination rates and inhibiting pollen tube elongation, which resulted in shortened siliques and decreased seed set, without affecting pollen morphology or viability. Transcriptome analysis of mature anthers revealed a transient and time-dependent transcriptional response, with the number of differentially expressed genes (DEGs) peaking at 8 h post-ABA treatment and markedly declining by 22 h. These DEGs were enriched in stress-response pathways (e.g., salt, cold, and dehydration), hormone signaling, and carbohydrate metabolism. Moreover, we identified 25 differentially expressed transcription factors and 16 pollen development and function-related genes, highlighting their key roles in ABA-mediated regulation. In parallel, 146 differentially expressed lncRNAs (DELs) were identified, which formed 144 *cis*-regulatory pairs with genes involved in ABA response and pollen tube growth, with their predicted targets enriched in pathways such as hormone and MAPK signaling, carbohydrate metabolism and stress response. *Trans*-regulatory analysis further revealed that these DELs co-expressed with DEGs in modules enriched for stress response, pollen development, and tube growth pathways. Notably, key pollen function genes showed strong co-expression with DELs, indicating that lncRNAs participate in ABA-induced transcriptional reprogramming that shifts metabolic resources from growth to defense, thereby suppressing pollen germination and tube elongation. Together, these findings elucidate a coordinated regulatory network involving mRNAs, lncRNAs and transcription factors roles in modulating ABA responses during pollen/anther development.

## 1. Introduction

In flowering plants, pollen possesses a sophisticated structure that not only protects the normal development of male gametes but also plays a crucial role in pollen-stigma recognition and fertilization processes [1,2]. Pollen development is a complex process, relying on the precise regulation of appropriate gene expression and function. It involves the dynamic, spatially and temporally coordination of diverse cells and tissues from both sporophytic and gametophytic origins [3,4,5]. This highly conserved process is initiated from anther cell division and differentiation, ultimately leading to male meiosis, germ cell formation, and pollen wall construction [6,7]. Within the anther, pollen mother cells undergo meiosis to produce tetrads of four haploid microspores enclosed by a callose wall. Following timely degradation of callose by tapetum-derived callase, the released microspores undergo asymmetric mitosis to form bicellular pollen grains containing a generative cell and a vegetative cell. The generative cell then undergoes a further mitotic division, yielding mature tricellular pollen grains composed of a vegetative cell and two sperm cells [3,8,9,10,11,12]. Concurrently, the pollen wall is elaborately constructed after microspore release, ensuring structural integrity and functional competence [13,14]. It is estimated that approximately 14,000 genes and 25,000 transcripts are expressed in the male gametophytes of the dicot plant model organism *Arabidopsis thaliana* and the monocot model rice (*Oryza sativa*), respectively [15,16,17]. Extensive genetic and transcriptomic studies have identified numerous protein-coding genes, especially transcription factors, involved in pollen/anther development, thereby laying the foundation for elucidating the regulatory mechanisms of male gametogenesis [15,18,19,20,21]

More recent investigations in model plants such as *Arabidopsis* and rice have pinpointed several non-coding RNAs (ncRNAs) that are vital for anther/pollen development and function [22]. Among ncRNAs, long non-coding RNAs (lncRNAs) are transcripts longer than 200 nucleotides (nt) that lack protein-coding potential [23]. They typically act as signaling molecules, decoys, guides, and scaffolds to regulate the expression of target genes, serving as key regulatory intermediates in complex biological networks [24]. An increasing number of studies have demonstrated that lncRNAs play important roles in pollen/anther development and male fertility [22,25,26,27]. For instance, in rice, the lncRNA *LDMAR* regulates photoperiod-sensitive nuclear male fertility. Elevated methylation in its promoter region suppresses transcription under long-day conditions, resulting in male sterility [28]. Another rice lncRNA, *PMS1T*, is preferentially expressed in young panicles and is processed by miR2118 to produce 21-nt phased secondary small interfering RNAs (phasiRNAs). These phasiRNAs accumulate preferentially in photoperiod-sensitive male sterile lines under long-day conditions, leading to male sterility [29]. In *Brassica campestris*, the pollen-specific lncRNA *BcMF11* regulates fertility and pollen development by ensuring proper tapetum degradation. Antisense transgenic plants of *BcMF11* exhibit aberrant tapetal degradation and microspore separation at the end of meiosis, ultimately resulting in the fusion of malformed microspores with tapetal remnants [30,31]. Two lncRNAs in *B*. *rapa*, *bra-eTM160-1* and *bra-eTM160-2*, act as endogenous decoys for miR160, thereby modulating the expression of the auxin response factor *ARF17* and contributing to pollen wall formation [32]. Overexpression of *lncRNA57811* in rice reduces pollen fertility and seed set [33]. In maize (*Zea mays*), the lncRNA *Zm401* activates *ZmMADS2* expression, which in turn regulates anther tapetum and microspore development [34]. In wheat (*Triticum aestivum*), the lncRNA *TaHTMAR* affects pollen fertility by upregulating *TaBBX25* and *TaOBF1*, both of which are expressed early during anther development [35]. Moreover, emerging evidence positions lncRNAs as key integrators of stress signals during pollen and anther development. The abundance of stress-induced lncRNAs in male reproductive organs, such as pollen, anthers, and tassels, suggests their involvement in mediating stress responses during male reproductive development [36,37,38]. In *Arabidopsis*, the heat shock factor HSFB2a, essential for both female and male gametophytic development, is regulated by a heat-inducible antisense lncRNA, *asHSFB2a*, revealing a feedback loop that links thermal stress response to reproductive fitness [39]. Similarly, overexpression of *lncRNA77580* in soybean not only enhances salinity tolerance and drought resistance at the seedling stage but also increases seed yield under water deficit during reproduction, underscoring the potential of lncRNAs to coordinate stress adaptation with reproductive output [40]. These findings highlight lncRNAs as crucial nodes in the regulatory networks that integrate environmental cues and developmental programs, thereby ensuring reproductive success under fluctuating conditions.

Beyond its regulation by a highly complex gene network, pollen development has been long recognized as being high susceptible to environmental stressors, such as temperature fluctuations, water deficit or excess, light intensity changes, as well as subtle changes in hormonal homeostasis [41,42,43]. Multiple plant hormones coordinately and tightly regulate the entire process of pollen development, among which abscisic acid (ABA) plays a dual role in balancing reproductive development and stress adaptation [44,45]. On one hand, ABA acts as a positive regulator in plant stress adaptation [46,47]. For instance, ABA enhances plant tolerance to salinity, drought, and acquired thermotolerance by regulating the expression of the heat shock factor *HSFA6b* [48]. Under drought stress, elevated ABA levels activate *ABI5* and other downstream transcription factors, orchestrating the expression of drought-responsive genes to strengthen drought resistance [49]. On the other hand, the intricate relationship between ABA and pollen/anther development has been increasingly recognized [50,51,52]. Fluctuations in ABA levels are sufficient to markedly impair pollen maturation, germination, and pollen tube growth [53,54,55]. SlNCED1, a key enzyme in ABA biosynthesis, is primarily present in ovules, stigmas, pollen/anthers, and vascular tissues, where it facilitates ABA formation. Both overexpression and suppression of *SlNCED1* disrupt normal pollen development [56]. Additionally, ABA plays a crucial role in mediating responses to environmental challenges that impact pollen. In wheat, under drought stress, ABA levels in pollen grains correlate strongly with male sterility and yield reduction [57,58,59]. In rice florets subjected to high-temperature stress, the concentration of ABA rise markedly, indicating its negative regulatory effects on reproductive process [60]. During rice pollen meiosis, drought stress reprograms gibberellin signaling and ABA catabolism-related pathways [61]. This dual role underscores the complexity of ABA signaling in mediating the trade-off between stress adaptation and reproductive success. However, due to the short duration and high complexity of pollen development, coupled with its internal localization within floral organs and the consequent challenges in sampling, the majority of how ABA impact pollen development and the underlying response mechanisms of pollen to these stresses are still hiding somewhere outside our realm of cognition [62,63,64].

Exogenous ABA was hypothesized to induce transcriptional reprogramming in *Arabidopsis* anthers through the coordinated regulation of specific lncRNAs and mRNAs, thereby impairing pollen development and/or function. To test this hypothesis, we treated the inflorescences of *Arabidopsis* with exogenous ABA and examined morphological and cytological changes in pollen. Subsequently, RNA-sequencing (RNA-seq) analysis was conducted to comprehensively identify and characterize differentially expressed protein-coding genes and lncRNAs in mature pollen before and after exogenous ABA treatment. As a highly sensitive and high-resolution transcriptomic tool, RNA-seq enables genome-wide profiling of gene expression dynamics with exceptional temporal resolution, making it particularly well-suited for capturing the transient and dynamic transcriptional changes induced by rapid hormone signaling such as that of ABA [65,66]. Additionally, it allows for the simultaneous detection of both coding and non-coding transcripts, facilitating the construction of regulatory networks that integrate multiple layers of gene regulation [67]. Our focus was on elucidating the regulatory relationships between lncRNAs and mRNAs, as well as their roles and significance in the response of pollen to exogenous ABA treatment. This study aims to provide insights into the molecular regulatory network underlying pollen development in flowering plants and their responses to hormones.

## 2. Results

### 2.1. Exogenous ABA Treatment Impacts Silique Development and Seed Set in Arabidopsis thaliana

**Figure 1:**
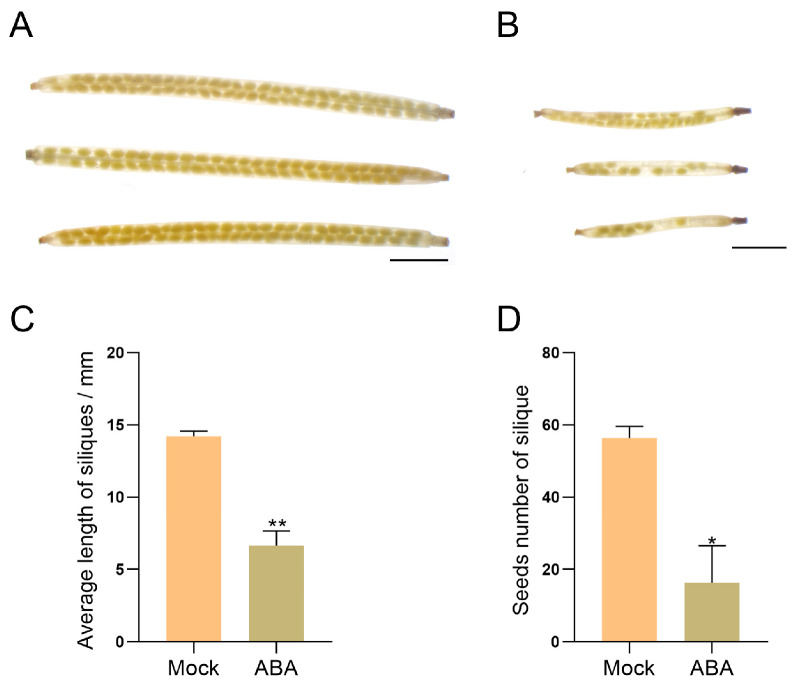
Observation of seed sets in siliques developed from ABA-treated *Arabidopsis* *thaliana* plants. (**A**) Siliques developed from mock control plants. (**B**) Siliques developed from 100 μmol/L ABA-treated plants. (**C**) Average length of siliques (*n* = 10). (**D**) Seed number of siliques (*n* = 10). Data were shown as mean ± SD. * *p* < 0.05 and ** *p* < 0.01. Scale bars = 2 mm.

To investigate the effects of exogenous ABA treatment on pollen development in *Arabidopsis*, plants were treated with 100 μmol/L ABA or mock controls and then transferred to optimal growth conditions. Following silique formation and seed maturation, silique length and seed set were measured. The results showed that exogenous ABA treatment significantly reduced silique length and seed set in *Arabidopsis* (Figure 1).

### 2.2. Exogenous ABA Treatment Affects Pollen Function Without Altering Pollen Development in Arabidopsis thaliana

**Figure 2:**
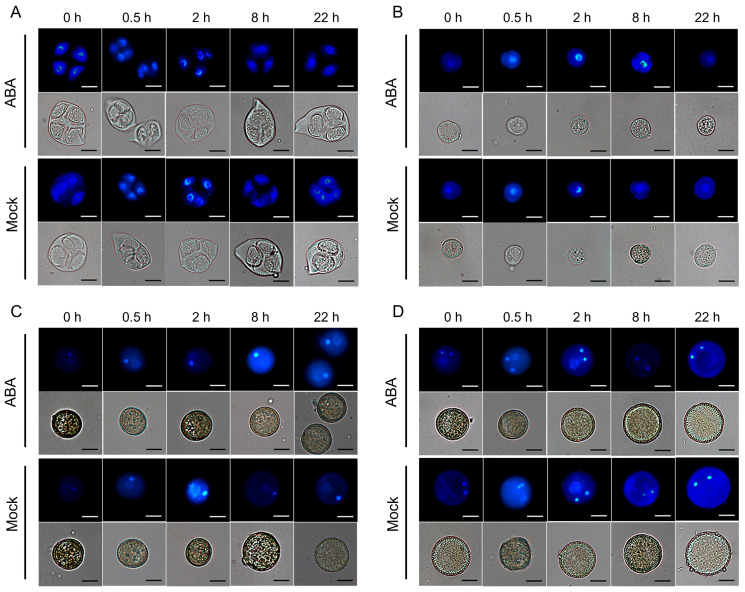
DAPI staining observation of developing microspores in *Arabidopsis thaliana* following exogenous ABA treatment. Microspores at different developmental stages were observed at 0, 0.5, 2, 8, and 22 h after treatment with 100 μmol/L ABA or mock solution. (**A**) Tetrad stage. (**B**) Uninucleate stage. (**C**) Bicellular stage. (**D**) Trinucleate stage. Scale bars = 10 μm.

**Figure 3:**
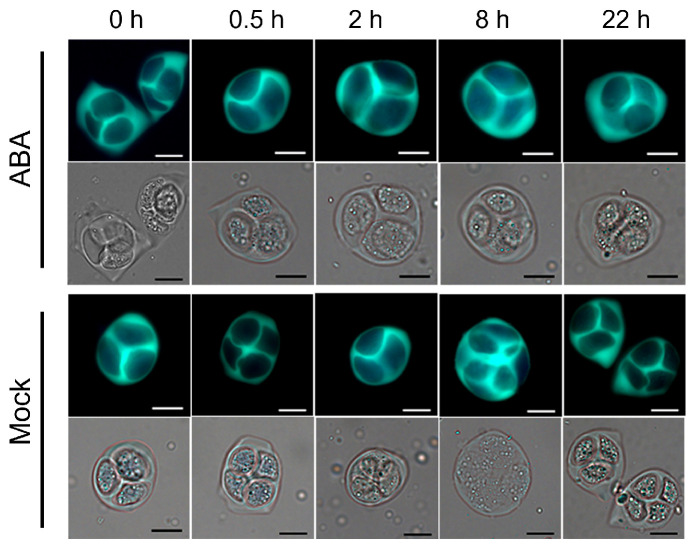
Aniline blue staining observation of tetrads in *Arabidopsis* *thaliana* following exogenous ABA treatment. Tetrads were observed at 0, 0.5, 2, 8 and 22 h after treatment with 100 μmol/L ABA or mock solution. Scale bars = 10 μm.

**Figure 4:**
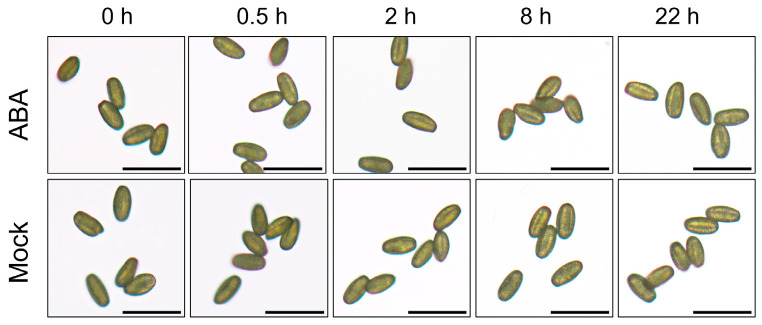
Morphological observation of mature pollen in *Arabidopsis* *thaliana* following exogenous ABA treatment. Mature pollen grains were observed at 0, 0.5, 2, 8, and 22 h after treatment with 100 μmol/L ABA or mock solution. Scale bars = 50 μm.

To elucidate how exogenous ABA treatment impacts silique development and seed set in *Arabidopsis*, instantaneous cytochemical staining was employed to observe pollen at different developmental stages. Staining with 4,6-diamidine-2-phenylindole (DAPI) revealed that ABA treatment failed to imprint on nuclei formation, as microspores observed at 0.5, 2, 8, and 22 h post-treatment at the tetrad, uninucleate, bicellular, and trinucleate stages showed no significant differences from the mock group (Figure 2). Furthermore, aniline blue staining of tetrads demonstrated consistent callose deposition in ABA-treated tetrads compared to the mock group (Figure 3). No obvious morphological differences were detected in mature pollen grains between ABA-treated and mock plants (Figure 4).

**Figure 5:**
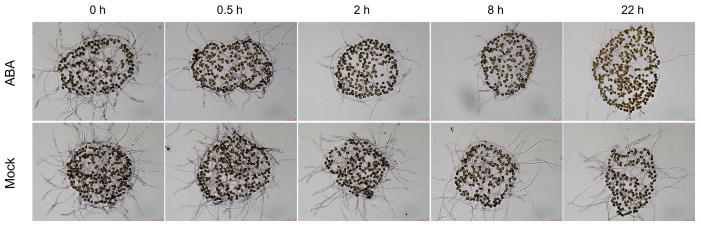
In vitro germination assay of mature pollen generated by ABA pre-treated *Arabidopsis* *thaliana* plants. Pollen germination were assessed at 0, 0.5, 2, 8, and 22 h after treatment with 100 μmol/L ABA or mock solution. Scale bars = 100 μm.

**Figure 6:**
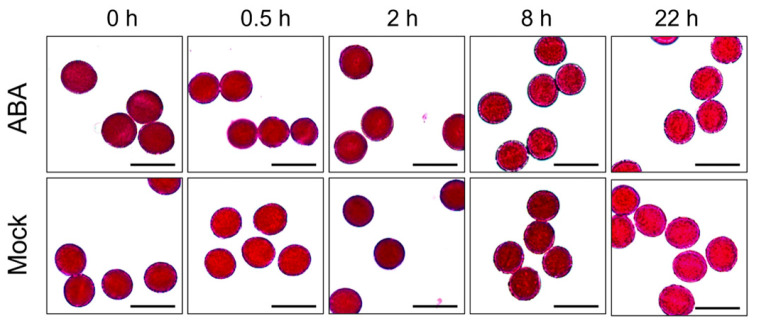
Alexander’s staining observation of mature pollen in *Arabidopsis* *thaliana* following exogenous ABA treatment. Mature pollen grains were stained at 0, 0.5, 2, 8, and 22 h after treatment with 100 mol/L ABA or mock solution. Scale bars = 20 μm.

However, it is worth noting that in vitro pollen germination and elongation were determined to be altered following ABA exposure. A reduced germination rate and inhibited pollen tube growth were observed compared to pollen from mock plants (Figure 5). Moreover, pollen germination rates exhibited a progressive decline over the 22 h observation duration following ABA treatment (Figure 5). However, pollen vitality, as assessed by the Alexander dye staining assay, remained not compromised by ABA treatment (Figure 6). These results indicate that ABA, in the concentration test, does not induce immediate or subsequent morphological changes in *Arabidopsis* developing pollen but rather impairs its function. This functional deficit likely underlies the observed shortening of siliques and reduced seed sets.

### 2.3. ABA Initiates a Transcriptional Cascade in Arabidopsis thaliana Anthers

#### 2.3.1. Differentially Expressed Protein-Coding RNAs

**Figure 7:**
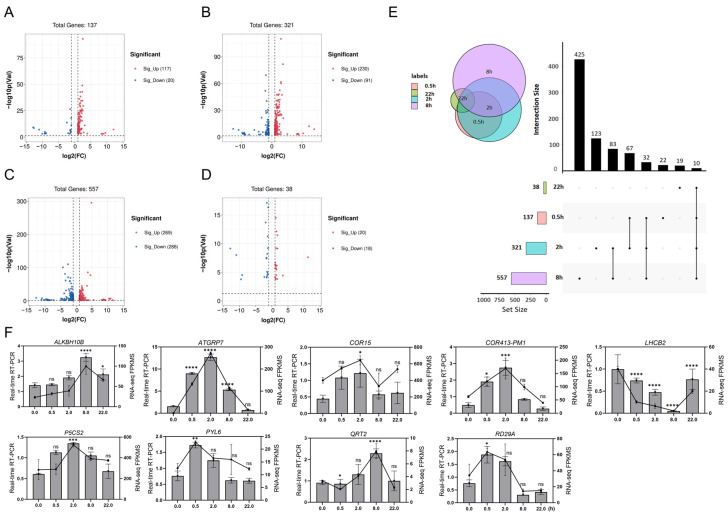
Transcriptional profiling of *Arabidopsis* *thaliana* anthers following ABA treatment. (**A**–**D**) Volcano plots illustrating differentially expressed genes (DEGs) in anthers at 0.5 (**A**), 2 (**B**), 8 (**C**), and 22 (**D**) h after ABA treatment, respectively, compared to the untreated control (UC_0 h). (**E**) Venn diagram showing overlap of DEGs across the four time points. (**F**) qRT-PCR validation of nine DEGs in ABA-treated anthers. Line graphs depict relative expression levels measured by qRT-PCR, while column graphs represent corresponding FPKM values from RNA-seq data. Data are presented as mean ± SD (*n* = 3). Statistical significance was determined by one-way ANOVA. * *p* < 0.05, ** *p* < 0.01, *** *p* < 0.001, and **** *p* < 0.0001.

To elucidate the molecular mechanisms underlying the ABA-induced decline in pollen germination rate and subsequent reduction in seed set, RNA-seq was performed on mature *Arabidopsis* anthers. Based on previous studies demonstrating that jasmonate-induced transcriptional cascades during anther development can be effectively captured within a 22 h time course [10], We harvested anthers from untreated stage 12 floral buds (designated as UC_0 h) and at 0.5, 2, 8, and 22 h after a single ABA application (designated as ABA_0.5 h, ABA_2 h, ABA_8 h, and ABA_22 h, respectively). This time-series design allows us to comprehensively profile the dynamic and transient gene expression changes triggered by ABA, from early signaling events to downstream regulatory responses. In total, 793 genes displayed altered expression levels relative to UC_0 h within the 22 h period following ABA treatment (Table S1). Specifically, 137, 321, 557, and 38 differentially expressed genes (DEGs) were identified in the comparisons of ABA_0.5 h vs. UC_0 h, ABA_2 h vs. UC_0 h, ABA_8 h vs. UC_0 h, and ABA_22 h vs. UC_0, respectively (Figure 7A–E). To verify the accuracy of the RNA-seq results, quantitative real-time PCR (qRT-PCR) analysis was conducted on nine DEGs. The strong consistency between the RNA-seq data and qRT-PCR validation (Figure 7F) confirmed the reliability of the transcriptomic profiling.

Transcriptomic profiling indicated an initial rise in both up- and down-regulated DEGs, peaking at 8 h (269 up and 288 down) (Figure 7A–C). However, this response was markedly attenuated by 22 h, with only 20 genes up-regulated and 18 down-regulated, representing a sharp decline from earlier time points and the lowest DEG count overall (Figure 7D). Furthermore, we identified 10 genes that were differentially expressed at all time points following ABA treatment relative to UC_0 h, including *CCR2*, *FKF1*, *JMJD5*, AT4G30650, *TSJT1*, AT4G30660, *ESL3.05*, *PUP8*, *UBQ5*, and AT2G05812. These findings reveal a dynamic yet transient transcriptional reprogramming in anthers following ABA treatment, characterized by an early, broad gene expression shift that substantially resolves within 22 h.

**Figure 8:**
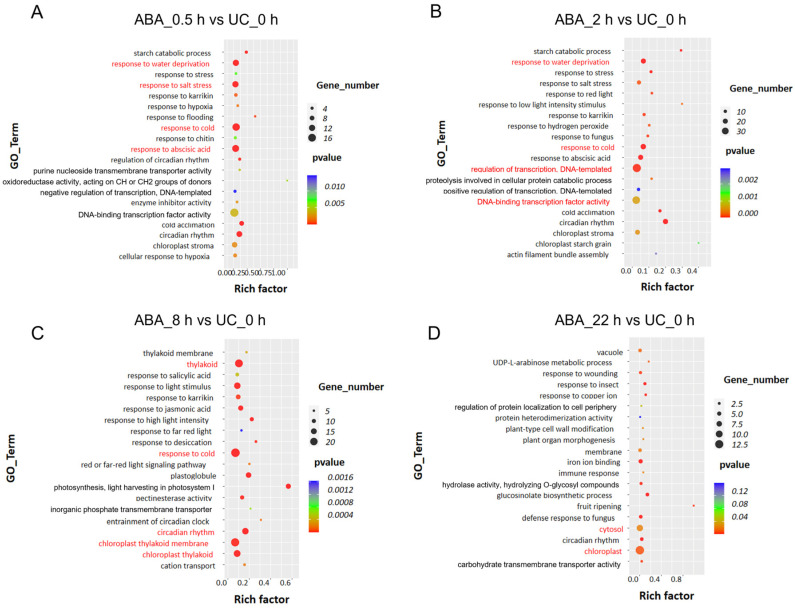
GO enrichment analysis of differentially expressed genes in *Arabidopsis* *thaliana* anthers following ABA treatment. (**A**–**D**) Enriched GO terms for differentially expressed genes identified at 0.5 (**A**), 2 (**B**), 8 (**C**), and 22 (**D**) h post-ABA treatment, compared to the untreated control (UC_0 h).

Gene Ontology (GO) and Kyoto Encyclopedia of Genes and Genomes (KEGG) enrichment analyses was subsequently performed to further characterize the functional dynamics of the observed transcriptional changes. GO analysis indicated that 94% of the DEGs (745 out of 793) were functionally annotated, with temporally distinct enrichment patterns across the treatment time course. At the early time following ABA treatment (0.5 and 2 h), genes associated with transcriptional regulation and DNA-binding factors were highly active (Figure 8A,B), indicating that transcriptional reprogramming represents an initial regulatory layer in response to external ABA. By 8 h post-ABA treatment, a sharp increase was observed in DEGs involved in stress responses, as well as in those related to chloroplasts, thylakoids, and thylakoid membranes (Figure 8C), suggesting that anthers initiated large-scale transcriptional regulation to cope with ABA signaling By 22 h, only 38 DEGs remained, reflecting that the transcriptional response had largely subsided; however, chloroplast-related genes continued to be highly expressed (Figure 8D), suggesting that this time point was not simply a decline in response but rather a shift toward adaptive or compensatory regulation centered on chloroplast function. Previous studies have reported that under water stress, chloroplast membrane disruption leads to ABA leakage and ABA level increase, underscoring a functional link between ABA and chloroplasts [68]. Long-term ABA exposure also suppresses thylakoid formation, chlorophyll biosynthesis, and the activities of RuBP carboxylase/oxygenase and PEP carboxylase [69,70,71]. Notably, the overarching theme of stress adaptation and defense activation persisted throughout the 22 h period (Figure 8). Among the top 20 most significantly enriched GO terms at 0.5 h, approximately 55% were concentrated in stress-response processes, such as salt stress, low-temperature stress, exogenous ABA stress, and dehydration stress (Figure 8A). Notably, low-temperature stress-related genes maintained high expression levels up to 8 h, suggesting potential crosstalk between ABA signaling and cold-response pathways (Figure 8). Together, these findings indicate that ABA treatment induces a temporally regulated, defense-oriented transcriptional program in anthers.

**Figure 9:**
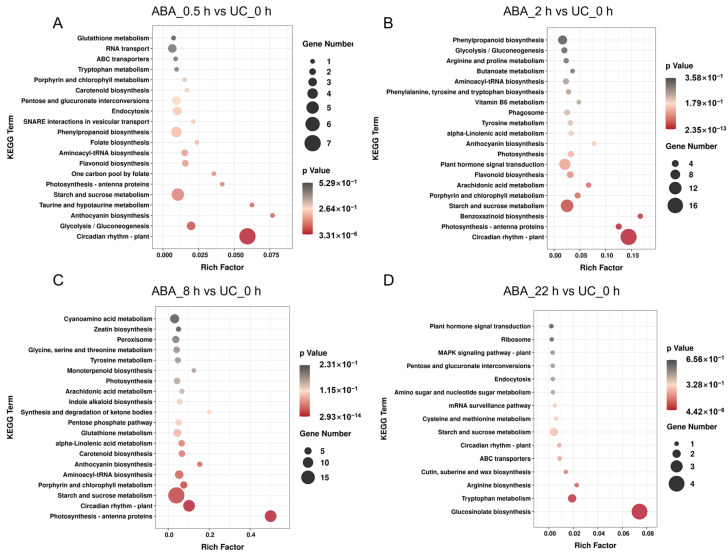
KEGG pathway enrichment analysis of differentially expressed genes in *Arabidopsis* *thaliana* anthers following ABA treatment. (**A**–**D**) Pathways were ranked by enrichment significance for differentially expressed genes identified at 0.5 (**A**), 2 (**B**), 8 (**C**), and 22 (**D**) h post-ABA treatment, compared to the untreated control (UC_0 h).

KEGG analysis further revealed time-specific reprogramming. During the early phase (0.5, and 2 h), DEGs were primarily enriched in pathways such as starch and sucrose metabolism and the plant circadian rhythm (Figure 9A,B). Beginning at 2 h, significant enrichment in plant hormone signaling pathways was observed (Figure 9B). By 8 h, the most diverse set of metabolic pathways was affected, including starch and sucrose metabolism, porphyrin and chlorophyll metabolism, photosynthesis-antenna proteins, anthocyanin biosynthesis, and several amino acid metabolic pathways (Figure 9C). In contrast, by 22 h, expression levels in most of these pathways had returned to near-baseline states, with only a limited number of pathways remaining deferentially expressed (Figure 9D). These findings highlight that ABA-induced transcriptional reprogramming follows a clear temporal progression, with the majority of metabolic adjustments occurring within the first 8 h and largely resolving by 22 h post-treatment.

#### 2.3.2. ABA-Responsive Transcription Factors

Transcription factors (TFs) have been implicated in reproductive development, including pollen germination, pollen tube growth, and seed development, by orchestrating the expression of downstream target genes in response to environmental signals [72,73,74,75]. To delineate the ABA-mediated transcriptional network governing these processes in *Arabidopsis* anthers, we identified 25 differentially expressed TFs following exogenous ABA treatment, comprising 15 up-regulated and 10 down-regulated genes (Table 1). Notably, with the exception of *ANAC029*, which showed significant down-regulation only after 8 h, all other TFs exhibited significant dysregulation early in the response, consistently within the first 8 h. This early and coordinated TF response highlights how ABA rapidly reshapes the transcriptional hierarchy to fine-tune pollen function and stress adaptation.

Among the up-regulated TFs, *MYB2*, *ANAC055*, and *ANAC075* are recognized for their roles in reproductive development and stress responses that impact pollen function. In *Arabidopsis*, *MYB2*, expressed in the tapetum during stages 5–11, is critical for tapetal programmed cell death and pollen viability. It directly regulates the expression of proteases *CEP1* and *βVPE*, thereby coordinating tapetal degradation and pollen maturation, processes essential for pollen germination and tube growth [76]. Under stress conditions, *MYB2* is upregulated and binds to the *RAX1* promoter to suppress its expression, thereby inhibiting axillary meristem formation. This enables plants to undergo a shorter vegetative development phase under adverse conditions [77]. *ANAC055* exhibits a co-expression with *ATAF1*, which has been shown to maintain high expression levels after heat stress and sustain elevated transcripts during prolonged recovery periods [78]. Under drought stress, *ANAC055* is transcriptionally activated and epigenetically regulated by CAU1 via H4R3sme2 histone methylation, providing a novel pathway for enhancing drought tolerance [79]. ANAC075 acts as a floral repressor; its loss-of-function up-regulates floral integrator genes and accelerates flowering [80]. ANAC075 also contributes to secondary cell wall biosynthesis [81]. Collectively, these TF dynamics illustrate how ABA reprograms transcriptional networks to regulate pollen germination and pollen tube growth, with specific TFs exerting their effects through the coordinated regulation of downstream target genes, thereby integrating developmental control with adaptive responses to environmental signals.

#### 2.3.3. DEGs Related to Pollen Development and Function

Drawing upon the published literature, we compiled a set of well-characterized genes involved in pollen development, germination and tube growth in *Arabidopsis*. Comparison with our RNA-seq data identified 16 DEGs involved in these processes following ABA treatment (Table 2). Among them, *YUC6* encodes a flavin monooxygenase associated with local auxin biosynthesis in microspores and regulates male gametophyte development [82]. *P5CS2* is expressed in developing microspores and mature pollen, participates in pollen development, and its homozygous mutant is lethal to microspore development [83]. *NAS3* encodes a nicotianamine synthase, and its loss-of-function results in arrested pollen development and failure of pollen germination and tube growth [84]. For genes regulating tapetal function and pollen wall formation, *MYB80* encodes a TF specifically expressed in the developing tapetum and microspores, regulating tapetal PCD [85]. *QRT2* is a polygalacturonase family member, essential for normal pollen separation [86]. *FST1* encodes a tapetum-specific flavonol sophoroside transporter involved in flavonol glycoside accumulation on the pollen surface; its loss-of-function leads to abnormal pollen wall composition [87]. *GLP10*, a DELLA-repressed stamen-enriched gene, encodes a germin-like protein that participates in stamen elongation and anther maturation, thereby supporting pollen development [88]. Regarding genes that regulate pollen germination and pollen tube growth, *EXL4* encodes pollen coat extracellular lipase 4 and mediates pollen hydration on the stigma to initiate germination [89]. *CSLA07* encodes a glycosyltransferase that catalyzes hemicellulose synthesis for the pollen tube wall; its heterozygous mutants exhibit pollen tube growth defects [90]. *LRX11* encodes a pollen-specific LRR-Extensin family protein that cooperates with homologous genes to maintain pollen tube wall integrity [91,92]. Both *PMEI1* and *PMEI2* encode pectin methylesterase inhibitor proteins enriched at the pollen tube tip, which regulate the stability of the pollen tube wall [93,94]. Additionally, among genes related to signal transduction and intracellular trafficking, *RABA4D* encodes a Rab GTPase of the RabA4 subfamily that regulates vesicle trafficking and polar growth at the pollen tube tip, facilitating rapid elongation of pollen tubes [95]. *CPK14* encodes a calcium-dependent protein kinase involved in calcium signaling at the pollen tube apex, and its activation induces depolarization of pollen tube growth [96]. *PRK4* encodes a pollen receptor-like kinase that is functionally redundant with *PRK5*; together, they cooperatively regulate actin dynamics and polar growth of pollen tubes, with the double mutant exhibiting pollen tube growth defects [97].

### 2.4. ABA-Induced LncRNAs Modulate Transcriptional Reprogramming in Arabidopsis thaliana Anthers

Recent decades of research have established that despite their low abundance, lncRNAs play critical roles in gene regulation throughout pollen and anther development, male fertility establishment, and stress adaptation [22]. They exert these effects through diverse mechanisms at transcriptional and post-transcriptional levels [98]. However, the understanding of how lncRNAs respond to hormone signals such as ABA and mediate the balance between pollen development/function and stress adaptation remains limited. Based on this, we further examined the expression dynamics of ABA-induced lncRNAs in mature *Arabidopsis* anthers. This analysis aimed to uncover whether and how ABA signaling reprograms the lncRNA-mediated regulatory landscape to coordinate pollen function.

#### 2.4.1. Identification and Characteristics of ABA-Responsive LncRNAs

**Figure 10:**
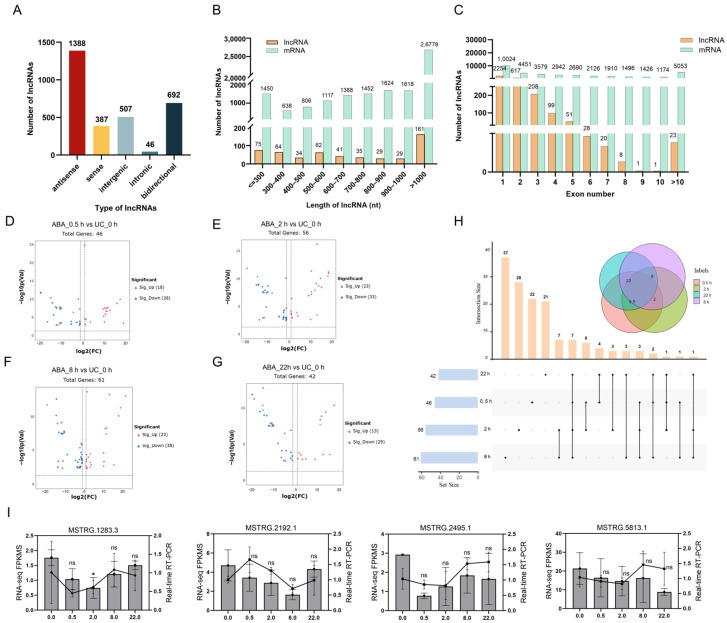
Transcriptome-wide analysis of differentially expressed lncRNAs in *Arabidopsis* *thaliana* anthers following ABA treatment. (**A**) Classification of identified lncRNAs. (**B**) Length comparison between lncRNAs and mRNAs. (**C**) Exon number comparison between lncRNAs and mRNAs. (**D**–**H**) Volcano plots of differentially expressed lncRNAs (DELs) in anthers at 0.5 (**D**), 2 (**E**), 8 (**F**), and 22 (**G**) h after ABA treatment, respectively, compared to the untreated control (UC_0 h). (**G**) Venn diagram showing overlap of DELs across the four time points. (**I**) qRT-PCR validation of four DELs in ABA-treated anthers. Line graphs depict relative expression levels measured by qRT-PCR, while column graphs represent corresponding FPKM values from RNA-seq data. Data are presented as mean ± SD (*n* = 3). * *p* < 0.05.

Our transcriptomic analysis revealed a total of 3310 lncRNAs expressed in mature *Arabidopsis* anthers (Table S2). Among them, 41.9% (1388) were antisense transcripts (NATs), 11.7% (387) were sense lncRNAs, 15.3% (507) were long intergenic lncRNAs (lincRNAs), 1.4% (46) were intronic lncRNAs (incRNAs), and 20.9% (692) were bidirectional lncRNA (Figure 10A). lncRNAs were notably shorter than protein-coding mRNAs, with lengths ranging from 201 to 9408; 79.9% (2646) fell to ≤ 1000 nt (Figure 10B). The exon number of lncRNAs was also lower than that of mRNAs, with 68.1% (2254) contained fewer than two exons, while the majority of mRNAs possessed more than five exons (Figure 10C).

Of the 3310 lncRNA identified, 146 were differentially expressed following ABA treatment (Table S3), of which 93 were previously annotated in the existing databases and 53 were novel. The number of differentially expressed lncRNAs (DELs) varied temporally, with 46, 56, 61, and 42 DELs detected at 0.5, 2, 8, and 22 h post-ABA treatment, respectively (Figure 10D–G, Table S3). qRT-PCR analysis of four DELs confirmed expression patterns consistent with the RNA-seq profiles (Figure 10I), further strengthens the reliability of our transcriptomic dataset. Venn analysis indicated that most DELs were specific to a single time point (Figure 10H). Among the altered transcripts, seven lncRNAs were differentially expressed across all four time points (Table S3). Four of these (MSTRG.19040, MSTRG.18558.4, MSTRG.2045.1, and MSTRG.13540.3) were specifically expressed in untreated anthers but became undetectable after ABA treatment, implying that ABA actively repress a subset of lncRNAs likely involved in maintaining basal pollen developmental programs. Their suppression by ABA may therefore facilitate the transition from developmental to stress-responsive states within anther. In contrast, the remaining three lncRNAs (MSTRG.231, MSTRG.6469, and MSTRG.18558.8) showed sustained up-regulation throughout the treatment period. Notably, MSTRG.231 and MSTRG.18558.8 exhibited particularly strong induction across all time points, suggesting they may represent core components of the ABA signaling pathway. Such lncRNAs could act as regulatory factors involved in establishing adaptive transcriptional programs in anthers under stress conditions.

#### 2.4.2. Extensive *cis*-Regulatory Relationships Between DELs and DEGs Induced by ABA in Mature *Arabidopsis* *thaliana* Anther

**Figure 11:**
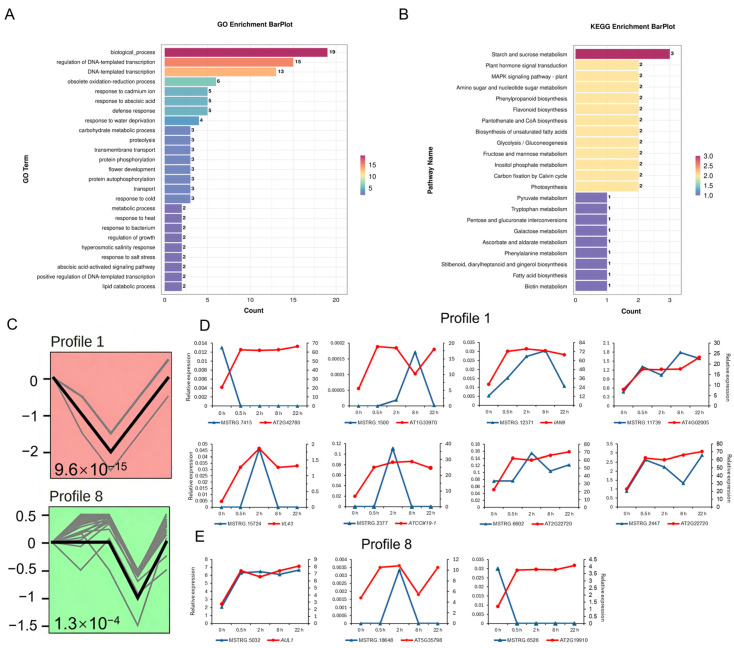
The *cis*-regulatory roles of lncRNAs during mature *Arabidopsis* *thaliana* anther response to ABA. (**A**,**B**) GO (**A**) and KEGG (**B**) enrichment analysis of targeted DEGs in DEL-DEG pairs. (**C**) Significantly clustered expression profiles of DELs obtained by STEM. Profile ordered based on the *p*-value significance. (**D**,**E**) Expression trends of DEL-DEG pairs from Profile 1 and Profile 8.

To further understand the involvement of lncRNAs during mature anther response to ABA treatment, we investigated potential *cis*-regulatory relationships by identifying target genes of DELs based on proximity. A total of 146 DELs were linked to 113 DEGs within a 100 kb window upstream and downstream on the chromosome. After consolidation, 144 unique DEL-DEG pairs were identified across four comparisons: ABA_0.5 h vs. UC_0 h (24 pairs), ABA_2 h vs. UC_0 h (48 pairs), ABA_8 h vs. UC_0 h (62 pairs), and ABA_22 h vs. UC_0 h (28 pairs) (Table S4). The majority of these lncRNA-target gene pairs (71.5%) were located within 100 kb of each other, while a smaller fraction (41 pairs) found within a 10 kb distance. GO enrichment analysis of these DEGs revealed a coherent molecular narrative centered on the induction of a potent abiotic stress response (Figure 11A), which directly explained the observed inhibition of pollen germination. The significant enrichment of GO terms related to ABA signaling and stress response indicates that DELs trigger a hormonal and physiological state antagonistic to pollen tube growth. This mechanistically supported by the concurrent dysregulation of growth-supportive processes, including carbohydrate metabolism, which are essential for pollen energy generation [99]. The KEGG pathway analysis further corroborates this shift, showing enrichment in ABA and mitogen-activated protein kinase (MAPK) signaling pathways alongside the central metabolic pathways like starch/sucrose metabolism and glycolysis (Figure 11B). Crucially, this stress reprogramming diverts resources toward defense-based secondary metabolism (e.g., phenylpropanoid biosynthesis) and away from the biosynthetic demands of rapid pollen tube elongation.

We further performed temporal expression profile clustering of all DELs using Short Time-series Expression Miner (STEM). A total of 20 expression profiles were generated (Table S5), and 2 profiles were identified as significantly enriched after filtration (Figure 11C and Table S6). Specifically, Profile 1 contained 99 DELs whose expression levels decreased to the minimum at 2 h post-ABA treatment and subsequently recovered gradually. In contrast, Profile 8 consisted of 38 DELs that maintained stable expression within the first 2 h after ABA treatment, followed by a gradual decline in expression abundance, reaching the lowest level at 8 h post-treatment before recovering. Target DEGs localized nearby DELs of these two profiles were analyzed for co-expression. Among the 13 DEL-DEG pairs identified, 9 and 2 showed positive and negative expression correlation, respectively (Figure 11D,E).

#### 2.4.3. LncRNA-Regulated Transcription Factors in Anther Response to ABA

The identification of several *cis*-regulated TFs among the predicted lncRNA target genes suggests a significant role for lncRNAs in mediating anther response to ABA by regulating the expression of these critical TFs (Table 3). Among these TF targets, SCL15 has been implicated as part of the HDA19 complex, where it represses the expression of embryonic-type genes in seedlings and contributes to the developmental transition from embryo to seedling. Notably, *SCL15* transcript levels surge under ABA and sucrose treatment [100]. Its homolog in rapeseed, BnSCL1, functions as an auxin-responsive protein known to interact with HDA19 [101]. WRKY57 directly targets the promoter sequence of *NCED3*; its overexpression demonstrably enhances drought tolerance and increases ABA content in *Arabidopsis* [102]. Similar benefits extend to rice, where *WRKY57* overexpression also improves salt and PEG tolerance [103]. *Arabidopsis ANAC029* has been found to be involved in cell division and expansion in anthers and petals, as well as in the regulation of leaf senescence [104,105]. Its homolog in tobacco, *NaNAC29*, participates in the defense response against tobacco brown spot disease by influencing the expression of *NaDLP1* and promoting leaf senescence [106]. *bHLH49* is associated with hormone signaling and cell elongation [107]. *AP1* is a floral meristem identity gene that integrates induction signals to initiate morphogenesis and coordinate floral organ development in conjunction with MADS-box TFs [108].

#### 2.4.4. ABA-Responsive and Flower Development-Related lncRNA–Target Gene Pairs

Two DEL-DEG pairs associated with ABA response were identified, involving the target DEGs *RCI2A* and *ABF1* (Table 3). *RCI2A* is induced not only by ABA but also by low temperature, dehydration, and salt stress [109]. *ABF1*, which is also induced by temperature fluctuations, is essential for winter seedling growth in *Arabidopsis* and plays a role in regulating seed dormancy and germination [110]. Furthermore, we identified three DEL-DEG pairs related to flower organ development (Table 3). Notably, we found that *RPOTM* a critical gene for pollen tube growth, female gametophyte development, and embryogenesis, was predicted to be dysregulated by lncRNA MSTRG.4503. *RPOTM* deficiency is known to cause delayed pollen tube growth and developmental abnormalities in ovules and embryos [111]. This dysregulation of *RPOTM*, targeted by lncRNA MSTRG.4503, provides a plausible mechanistic explanation for the observed disruption of pollen germination following ABA treatment, although further experimental validation is required.

#### 2.4.5. *Trans*-Regulatory Roles of DELs in Mature Anther Response to ABA

**Figure 12:**
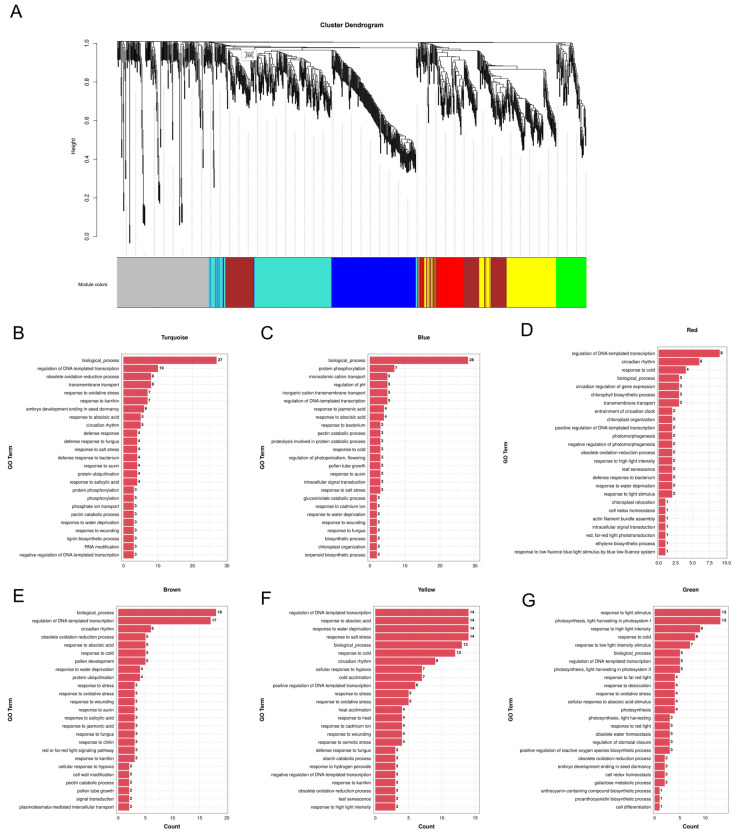
Weighted gene correlation network analysis (WGCNA) of differentially expressed lncRNAs and differentially expressed genes. (**A**) The cluster dendrogram constructed by WGCNA. (**B**–**G**) The top 25 biological processes of GO annotation of differentially expressed genes in turquoise (**B**), blue (**C**) red (**D**), brown (**E**), yellow and (**F**) green (**G**) modules.

Beyond their known *cis*-regulatory roles, lncRNAs have also been demonstrated to influence protein-coding gene expression through *trans*-acting mechanisms [112,113]. To further investigate the function of these DELs in anther response to ABA, a weighted gene co-expression network analysis (WGCNA) integrating both DEGs and DELs was performed. Ultimately, six distinct co-expression modules were identified, each assigned a unique color (Figure 12A and Table S7). Based on the GO annotation results across all six modules, DEGs are consistently and significantly enriched in transcriptional regulation processes (e.g., regulation of DNA-templated transcription), hormone responses (e.g., response to ABA and jasmonic acid), and stress responses (e.g., response to cold and salt stress) (Figure 12B–G). These enrichments collectively reinforced that ABA treatment rapidly elicits a lncRNA-mediated genome-wide transcriptional reprogramming in anthers, characterized by enhanced regulation of gene expression, hormone signaling, and stress adaptation pathways.

#### 2.4.6. *Trans*-Regulated Genes Essential for Pollen Development and Tube Growth

Notably, DEGs in the MEblue module were specifically annotated to pollen tube growth, while the MEbrown module contained 135 DEGs enriched in pathways related to pollen development and pollen tube growth (Figure 12C–E). These modules encompassed several core genes with well-characterized roles in regulating pollen development and pollen tube growth in *Arabidopsis* (Table S7). *FST1*, a tapetum-specific transporter, is essential for the translocation and accumulation of flavonol sophorosides from the tapetum to the pollen surface [87]. *AtSTP11* is a high-affinity H^+^-monosaccharide transporter specifically expressed in the pollen tube plasma membrane, furnishing monosaccharides essential for rapid pollen tube elongation and sustaining carbon nutrient homeostasis [114]. *PPME1* and *PMEI2* act synergistically to maintain the highly esterified and extensible state of pectin at the pollen tube tip, ensuring rapid polar growth [93]. *CHX21* and *CHX23*, functionally redundant K^+^/H^+^ exchangers in pollen tubes, mediate pistil signal perception and polar reorientation by regulating intracellular local cation balance and pH homeostasis [115]. *PRK4* encodes a receptor-like kinase that, in conjunction with pollen-specific *NET2A* (a member of the NET family), regulates polar growth and pistil signal response in pollen tubes [97]. *P5CS2*, a rate-limiting enzyme in proline synthesis, is required for pollen development and male fertility, driving de novo proline synthesis in microspores and mature pollen, a process irreplaceable by exogenous proline transport [83]. *UGE3*, a UDP-glucose 4-epimerase 3, is markedly upregulated during pollen germination and tube growth. It catalyzes the conversion of UDP-glucose to UDP-galactose to provide key precursors for pollen tube cell wall biosynthesis, and acts synergistically with *UGE2* to ensure pollen development and plant fertility [83]. *NAS3* mediates the chelation and translocation of metal ions via nicotianamine synthesis, maintaining metal homeostasis during pollen development and tube growth. It also collaborates with other family members to promote efficient iron unloading from the phloem to floral reproductive sinks such as anthers, acting as a key gene for nicotianamine-modulated male fertility in *Arabidopsis* [84].

## 3. Discussion

### 3.1. Exogenous ABA Specifically Impairs Pollen Function in Arabidopsis thaliana

ABA is a multifunctional phytohormone that regulates diverse aspects of plant growth and development [58,116]. Its role in reproductive development has been extensively documented, particularly in pollen and anther maturation. In cereals, disrupted ABA homeostasis within pollen grains is strongly correlated with male sterility and yield reduction [51,57,58,59,61,117]. The central role of ABA is corroborated by experimental perturbation, where exogenous ABA application during microspore development induces pollen abortion, reduces seed set, and alters the expression of cell wall invertase and monosaccharide transporter genes [51]. Despite these advances, the precise molecular mechanisms through which ABA influences pollen/anther development and the full scope of its downstream regulatory networks remain to be fully elucidated.

Previous studies have established that ABA exerts dose-dependent effects on plant growth and fertility. For instance, lower concentrations (e.g., 50 μmol/L) promote the somatic embryo development in soybean [118,119], whereas higher concentrations (e.g., 100 μmol/L) significantly inhibit plant growth and reproductive development in *Arabidopsis* [120]. Similarly, in rice, 100 μmol/L ABA markedly increases the proportion of spikelets with non-closed glumes [121]. Exogenous ABA has also been reported to reduce pollen viability dose-dependently, with notable inhibitory effects at 100 μmol/L and higher concentrations [117]. Moreover, this concentration effectively mimics ABA levels observed under abiotic stresses, enabling the exploration of ABA-mediated stress responses in reproductive tissues [122]. Therefore, 100 μmol/L ABA was used for exogenous treatment in this study. Phenotypic analysis in this study revealed that exogenous ABA treatment led to abnormal silique development and a significant decrease in seed set in *Arabidopsis* (Figure 1), consistent with previous reports [123]. However, cytological examinations showed that callose wall deposition during tetrad stage and nuclear development across all stages remained unaffected (Figure 2 and Figure 3), and mature pollen morphology was normal (Figure 4). Notably, in vitro pollen germination assays demonstrated that both pollen germination rate and pollen tube elongation were significantly inhibited in ABA-treated plants, whereas pollen morphology and viability remined unaffected (Figure 4, Figure 5 and Figure 6). This specific impairment of pollen function likely constitutes the direct cause of the observed silique malformation and reduced seed set in *Arabidopsis* following ABA treatment. It is worth noting that the effects of ABA on pollen are not universally inhibitory. Several studies have reported that ABA can stimulate pollen tube growth both in vitro and in vivo [124,125]. For instance, exogenous ABA at concentrations ranging from 0.1 to 100 μmol/L stimulated pollen germination and tube growth in *Petunia hybrida* [125]. In barley (*Hordeum vulgare*) anther cultures, ABA application enhanced microspore viability and reduced apoptosis-like features [126]. These seemingly divergent outcomes underscore that ABA can exert both stimulatory and inhibitory effects on pollen depending on concentration, developmental stage, and species.

### 3.2. ABA Induces a Transient Transcriptional Reprogramming That Disrupts Pollen Function

RNA-seq analysis further elucidated the molecular dynamic of *Arabidopsis* anthers in response to exogenous ABA treatment. The number of DEGs peaked at 8 h post-treatment and declined markedly by 22 h, indicating a transient transcriptional reprogramming characterized by rapid induction followed by gradual attenuation (Figure 7A–E). This temporal pattern contrasts with that observed in jasmonate-treated anthers, where induced genes increased progressively from 31 at 0.5 h to 1577 at 22 h [10]. The distinct kinetics suggest different regulatory mechanisms underlying these two hormone signaling pathways during anther development. Jasmonate triggers a sustained transcriptional cascade that continues to expand through 22 h, likely driving progressive developmental processes such as anther dehiscence and filament elongation [10]. In contrast, ABA elicits a more transient response, peaking at 8 h followed by rapid attenuation by 22 h. This transient pattern may reflect ABA’s role as a rapid stress signal triggering immediate adaptive responses, rather than a sustained developmental program. Interestingly, despite the overall decline in DEGs by 22 h, chloroplast-related genes among the ABA-responsive DEGs remained highly expressed. This sustained expression suggests a shift to an adaptive and compensatory response centered on chloroplast function, which is critical for maintaining cellular homeostasis under stress conditions [127,128].

GO and KEGG enrichment analyses showed that early DEGs (0.5–2 h) were enriched in transcriptional regulation and DNA-binding functions. By 8 h, DEGs involved in stress response and chloroplast-related processes became prominent (Figure 8 and Figure 9). Notably, genes related to cold stress response maintained highly expressed until 8 h, suggesting potential crosstalk between ABA and cold response signaling. This observation aligns with previous reports where the *fry1* mutant exhibits enhanced sensitivity to both ABA and cold stress [129]. Prior studies in rice and chickpea anthers have established that low-temperature stress triggers a marked accumulation of ABA [51,130]. ABA accumulation under cold stress plays a dual role, enhancing stress tolerance while simultaneously impairing pollen development and leading to male sterility. This process is regulated by a balance between ABA biosynthesis (via *NCED/ZEP*) and catabolism (via *ABA8′OH*), with tolerant varieties often maintaining lower ABA levels through efficient inactivation [92,93]. By 22 h, most metabolic pathways returned to near-baseline levels, while the MAPK cascade signaling pathway became significantly enriched (Figure 9). MAPKs are known to function as convergence nodes for oxidative stress signals [131]. Concurrently, we observed differential expression of genes associated with oxidative stress response in our transcriptomic data (Table S1). These findings collectively corroborated previous studies demonstrating that ABA, through modulation of ROS levels, participates in the regulation of tapetal programmed cell death, pollen development, and pollen germination [117,132]. Together, the temporal dynamic of DEGs reflect the time-specific nature of the anther’s transcriptional response to ABA. Notably, among the DEGs, 16 genes directly related to pollen development were identified, spanning key processes such as pollen morphogenesis, pollen tube growth, and signal transduction (Table 2), including *YUC6* [82], *P5CS2* [83], and *NAS3* [83]. The differential expression provided a molecular basis for the observed pollen germination defects.

### 3.3. Transcription Factors as Key Hubs in ABA-Mediated Pollen Function Disruption

TFs serve as key hubs in ABA signal transduction. In this study, 25 TFs were differentially expressed upon ABA treatment, among which *MYB2*, *ANAC055*, and *ANAC075* have previously been implicated in reproductive development and stress responses (Table 1). Here, ABA-induced upregulated of *MYB2* may accelerate tapetal PCD, thereby indirectly impairing pollen function. Additionally, *MYB2* represses *RAX1* to modulate the transition from vegetative to reproductive growth; its mis-expression could therefore disturb the balance of reproductive development [77]. *ANAC055* and *ANAC075* are involved in drought response and flowering-time regulation, respectively [78]. Their differential expression implies that ABA may coordinate stress adaptation with reproductive development through the modulation of NAC-family TFs.

### 3.4. LncRNAs as Emerging Regulators in Anther Response to ABA

Beyond TFs, lncRNAs are emerging as important regulators. While lncRNAs have been partially characterized for their functions in ABA responses during vegetative growth [17,133], their roles in reproductive organs remain less defined. Transcriptomic profiling in this study identified 3310 lncRNAs expressed in mature anthers of *Arabidopsis*. The majority of these lncRNAs were shorter than 2 kb and harbored fewer than five exons, consistent with the typical structural features of plant lncRNAs. Notably, the abundance of lncRNAs was lower in mature anthers compare to earlier developmental stages, such as the uninucleate, bicellular and tricellular pollen stages [134], mirroring the general transcriptional downregulation trend observed for protein-coding genes as anther mature [134,135].

*Cis*-target gene prediction identified 144 DEL-DEG pairs (Table S4), with target genes enriched in stress response, floral organ development, and hormone signaling (Figure 11A,B). Notably, specific DEL-DEG pairs directly related to ABA signaling (*RCI2A* and *ABF1*) and floral development (*RPOTM*, *AP1*, and *ANAC029*) were identified (Table 3) [109,110,111]. The discovery of these lncRNA–target gene pairs, particularly the *cis*-regulation of *RPOTM* by lncRNA MSTRG.4503, provides a plausible mechanistic link for the observed ABA-induced inhibition of pollen germination, although this requires further experimental validation. Collectively, these findings offer direct molecular clues for the role of lncRNAs in ABA-regulated reproductive development. Furthermore, the identification of several TFs (e.g., *SCL15*, *WRKY57*, *ANAC029*, *bHLH49*) among the predicted lncRNA target genes (Table 3) indicates that lncRNAs may exert their influence by *cis*-regulating key transcriptional regulators [136], thereby participating in the complex transcriptional reprogramming of anthers in response to ABA.

Temporal clustering of DELs using STEM revealed two significantly enriched expression profiles (Profile 1 and Profile 8) (Figure 11C). The distinct temporal expression characteristics of DELs in these profiles are consistent with the established concept that plant ncRNAs display time-dependent expression profiles in response to environmental signals. For instance, drought-responsive lncRNAs in rice exhibit distinct temporal expression features under abiotic stresses including drought and ABA [137], reinforcing the time-dependent nature of lncRNA-mediated stress responses. WGCNA further identified six co-expression modules containing both DELs and DEGs, which directly confirms their extensive co-expression under ABA treatment. Studies on waterlogging tolerance in *Secale cereale* L. have demonstrated that lncRNAs and mRNAs form co-expression modules to mediate abiotic stress responses [138], while research on pollen development in *Arabidopsis* has revealed that such co-expression network exerts key regulatory functions [134]. These findings indicate that lncRNA–mRNA co-expression represents a conserved regulatory strategy in plants. Our results suggest that DELs and DEGs co-expressed within the same module may have direct or indirect regulatory relationships, providing valuable insights for future dissection of the molecular network underlying ABA-regulated pollen function.

Based on these findings, we propose an integrated model for ABA-mediated pollen dysfunction in *Arabidopsis*. Upon ABA treatment, a transient transcriptional reprogramming is initiated, characterized by the rapid activation of stress-responsive transcription factors (e.g., *MYB2*, *ANAC055*, *ANAC075*), which serve as key hubs linking ABA signaling to downstream regulatory cascades. These TFs orchestrate the differential expression of genes involved in metabolic reprogramming, including hormone signaling, sugar metabolism, and chloroplast function, redirecting cellular resources toward stress adaptation. Concurrently, lncRNAs act as fine-tuners of this response through *cis*- and *trans*-regulatory networks, modulating the expression of ABA signaling components (e.g., *RCI2A*, *ABF1*) and pollen development-related genes (e.g., *RPOTM*). The co-expression patterns revealed by WGCNA further support that lncRNAs and mRNAs function coordinately to mediate the anther’s response to ABA. Ultimately, these multi-layered regulatory events converge on the disruption of core pollen developmental processes, including pollen germination and tube growth, leading to reduced seed set. This model advances our understanding of stress response in flowering plants and provides potential targets for breeding stress-resilient crops.

### 3.5. Future Perspectives

Future studies should prioritize the functional validation of core lncRNAs and their target genes implicated in ABA-mediated pollen dysfunction. This can be accomplished through the generation of knockout or overexpression lines of key lncRNAs, coupled with molecular assays such as EMSA, dual-luciferase reporter assay, and ChIP-qPCR to delineate their *cis*- and *trans*-regulatory mechanisms. qRT-PCR validation and in situ hybridization of candidate gene expression patterns will further substantiate the link between transcriptomic changes and phenotypic outcomes. Moreover, integrating multi-omics approaches (e.g., proteomics, metabolomics) alongside genetic crosses with ABA signaling mutants will facilitate a comprehensive dissection of the regulatory cascades underlying ABA-mediated pollen development inhibition. These efforts will systematically improve our understanding of the molecular basis underlying the trade-off between reproductive development and stress adaptation in plants, and will inform the breeding of stress-tolerant, high-yielding crop varieties.

## 4. Materials and Methods

### 4.1. Plant Material and Growth Conditions

*Arabidopsis thaliana* (Columbia-0, Col-0) plants were cultivated in a substrate mixture of peat, vermiculite, and perlite (3:2:1, *v*/*v*) under controlled environmental conditions: 22 °C, light intensity of 24,000 lx, 16 h light/8 h dark photoperiod, and 60% relative humidity.

### 4.2. ABA Treatment

For ABA treatment, 35-day-old *Arabidopsis* plants with uniform growth were selected one day prior to treatment. Lateral branches were removed, and all open flowers and whitish buds on the main inflorescence were excised. On the day of treatment, inflorescences bearing floral buds from stage 5 (pollen mother cell stage) to stage 12 (trinucleate stage) were submerged for 1 min in 100 μmol/L ABA solution, using 0.01% Silwet L-77 as the corresponding mock control and surfactant. Inflorescences were submerged in each solution for 1 min. Following treatment, all plants were returned to standard growth conditions.

### 4.3. Pollen Extraction

At each time point (0, 0.5, 2, 8, and 22 h post-ABA or mock treatment), approximately 300 unopened floral buds were collected in triplicate for each condition. Developing microspores at the tetrad, uninucleate, bicellular, and trinucleate stages were extracted by adding 0.3 mol/L mannitol solution and 5 mm grinding beads (Jingxin, Shanghai, China, Cat. No. JX-YG0117) to the floral buds in 2 mL centrifuge tubes. The samples were then ground at 20 Hz for 60 s using an automatic grinding mill (Jingxin, Shanghai, China, JXFSTPRP-24L). The resulting suspension was sequentially filtered through 100 µm and 41 µm nylon membranes. The filtered pollen was pelleted by centrifugation at 7000 rpm for 5 min.

### 4.4. Microscopic Staining Observation

To investigate callose deposition, microspores at the tetrad stage were harvested and mounted on a glass slide. A 20 μL volume of 0.1% aniline blue solution was applied to the tetrads. After a 5 min incubation, a coverslip was affixed. Stained tetrads were observed and imaged using a fluorescence microscope (DM3000 LED, Leica, Wetzlar, Germany) under UV filter with excitation at 340–380 nm and emission at 420–485 nm.

For assessing nuclear development, DAPI staining was performed. Extracted microspores at the tetrad, uninucleate, bicellular, and trinucleate stages were gently dispersed into 20 μL of DAPI staining solution. After a 5 min incubation in the dark, the stained nuclei were visualized and documented under a fluorescence microscope (DM3000 LED, Leica, Wetzlar, Germany) under UV filter with excitation at 340–380 nm and emission at 420–485 nm.

Pollen viability was assessed using Alexander’s staining. Mature pollen grains were mixed with 20 μL of Alexander’s staining solution (1 mL 75% ethanol, 100 μL 1% malachite green, 500 μL 1% acid fuchsin, 50 μL 1% Orange G, 400 μL glacial acetic acid, and 2.5 mL glycerol). After a 5 min incubation, the stained pollen grains were examined and photographed under a bright-filed microscope (DM3000 LED, Leica, Wetzlar, Germany).

### 4.5. In Vitro Pollen Germination Assay

A solid pollen germination medium was prepared by combining 400 μL of pollen germination solution (0.01% boric acid, 5 mmol/L CaCl_2_, 5 mmol/L KCl, and 1 mmol/L MgSO_4_, each at 100 μL), 1 g sucrose, 7.5 mL distilled water, and 0.05 g agarose. The pH was adjusted to 7.5 using 0.1 mol/L NaOH. The mixture was heated in a microwave to dissolve the agarose, and 200 μL was aliquoted and spread evenly onto glass slides to solidify. For in vitro germination assay, mature anthers were pressed onto the solidified medium. Slides were placed in a Petri dish lined with moistened cheesecloth and incubated in a growth chamber used for 5 h. Pollen germination and pollen tube growth were then observed and imaged under a fluorescence microscope (DM3000 LED, Leica, Germany).

### 4.6. Silique Observation

Following ABA treatment, *Arabidopsis* plants were transferred to optimal growth conditions. Once siliques had developed and seeds matured, at least 10 siliques per plant were randomly selected for observation. The siliques were decolorized by fixation overnight in a solution of acetic acid: ethanol (1:9, *v*/*v*), following by immersion in 90% ethanol for 1 h at room temperature, then 70% ethanol and storage at 4 °C overnight. The siliques were then observed and photographed under a stereomicroscope (SMZ800N, Nikon, Tokyo, Japan). Silique length and seed set rate were statistically analyzed.

### 4.7. RNA-Seq and Data Analysis

Following ABA treatment, mature anthers on the main inflorescences were collected at 0, 0.5, 2, 8, and 22 h for RNA-Seq library construction. Total RNA was extracted using Trizol reagent (Invitrogen, Carlsbad, CA, USA), with three biological replicates. Strand-specific RNA-seq libraries (fr-firstrand) were constructed using NEBNext® Magnesium RNA Fragmentation Module (New England Biolabs, Ipswich, MA, USA, Cat. No. E6150S) followed by ribosomal RNA depletion with the Ribo-Zero Gold rRNA Removal Kit (Illumina, San Diego, CA, USA, Cat. No. MRZG12324). Subsequently, sequencing was performed on the Illumina Novaseq™ 6000 platform by Hangzhou Lianchuan Biotechnology Co., Ltd. (Hangzhou, China), with a read length of 2 × 150 bp (PE150). Raw reads were quality-checked and filtered using Cutadapt (v1.9) to remove low-quality sequences. Clean reads were aligned to the *Arabidopsis* reference genome using Hisat2 (v2.2.1). Gene expression levels were quantified using fragments per kilobase of transcript per million mapped reads (FPKMs) for visualization of expression patterns. Differential expression analysis was conducted using the edgeR package (v3.22.5) based on raw read counts. Meanwhile, StringTie (v2.1.6) was used to assemble reads aligned to the genome, and known mRNAs and transcripts shorter than 200 bp were excluded. The remaining transcripts were analyzed for coding potential using CPC (Coding Potential Calculator, v0.9-r2) and CNCI (Coding–Noncoding Index, v2.0) software. Transcripts with no coding potential were identified as lncRNAs. Genes and lncRNAs with an absolute log2 fold change (|log2FC)|) ≥ 1 and a q-value < 0.05 in at least one comparison (ABA_0.5 h vs. UC_0 h, ABA_2 h vs. UC_0 h, ABA_8 h vs. UC_0 h, or ABA_22 h vs. UC_0 h) were considered as differentially expressed. GO enrichment and KEGG pathway enrichment analyses were performed on the DEGs.

### 4.8. RNA-Seq Data Validation by qRT-PCR

To validate the reliability of RNA-seq data, the expression patterns of nine randomly selected DEGs and four DELs were assessed using qRT-PCR. *Tubulin-4* (*Tub4*) was used as the reference gene. Primers for the candidate genes were designed using Primer Premier 5 software (Table S8). The qRT-PCR reaction was performed in a 20 µL system containing 10 µL of SYBR Green Master Mix (Monad, Wuhan, China, Cat. No. MQ10101), 1 µL of each forward and reverse primer, 1 µL of cDNA template, and 7 µL of ddH_2_O. Reactions were performed on a BioRad CFX Connect qRT-PCR Detection System (Bio-Rad, Hercules, CA, USA) with the following cycling conditions: initial denaturation at 95 °C for 30 s, followed by 40 cycles of 95 °C for 10 s, 60 °C for 30 s, and 72 °C for 30 s. The experiment was conducted with three biological replicates and three technical replicates. Relative expression levels were calculated using the 2^−ΔΔCt^ method.

### 4.9. STEM Analysis

To characterize the dynamic expression patterns of DELs during mature anther response to ABA) treatment, expression profile clustering was conducted using STEM (v1.3.11). Expression profiles were grouped into 20 predefined clusters, and only those meeting a significance threshold of *p* < 0.05 were considered enriched.

### 4.10. WGCNA

The WGCNA was conducted using the R package to perform hierarchical clustering analysis on DEGs and DELs, thereby establishing a co-expression network between lncRNAs and mRNAs. DEGs and DELs exhibiting similar expression patterns were clustered into distinct co-expression modules. Each module was assigned a unique color label, and its module eigengene was defined as the first principal component of the expression matrix for each respective module.

### 4.11. Statistical Analysis

All experiments were performed with at least three biological replicates. Statistical differences between groups were assessed using t-test or one-way ANOVA with GraphPad Prism version 9.0. *p*-values < 0.05 were considered statistically significant.

## Figures and Tables

**Table 1:** Differentially expressed transcription factors in anthers following ABA treatment in *Arabidopsis thaliana*.

Gene Name	Gene ID	log_2_ FC				Description
		ABA_0.5 h vs. UC_0 h	ABA_2 h vs. UC_0 h	ABA_8 h vs. UC_0 h	ABA_22 h vs. UC_0 h	
*NAC047*	AT3G04070	1.06	1.42	0.52	−0.36	NAC domain containing protein 47
*PLATZ2*	AT1G76590	1.18	1.28	0.62	−0.21	PLATZ transcription factor family protein
*AtPLATZ12*	AT5G46710	1.28	0.97	−0.08	−0.18	PLATZ transcription factor family protein
*PAP8*	AT1G21600	0.58	1.07	1.22	−0.33	Plastid transcriptionally active 6
*PTF1*	AT3G02150	−0.17	0.18	1.23	0.31	Plastid transcription factor 1
*NAC046*	AT3G04060	1.24	1.18	0.64	0.65	NAC domain containing protein 46
*WLIM2a*	AT2G39900	0.91	1.14	0.43	−0.31	GATA type zinc finger transcription factor family protein
*AtPLATZ4*	AT1G43000	0.25	0.85	1.14	0.06	PLATZ transcription factor family protein
*ATWLIM1*	AT1G10200	0.93	1.09	0.76	−0.23	GATA type zinc finger transcription factor family protein
*MYB2*	AT2G47190	0.67	1.08	0.20	0.43	MYB domain protein 2
*HRA1*	AT3G10040	1.07	0.36	−0.28	0.39	Sequence-specific DNA binding transcription factors
*ANAC055*	AT3G15500	0.87	1.03	0.61	0.00	NAC domain containing protein 3
*ANAC075*	AT4G29230	0.45	0.91	1.02	−0.41	NAC domain containing protein 75
*ANAC043*	AT2G46770	0.40	0.49	1.02	0.26	NAC domain transcriptional regulator superfamily protein
*WRKY17*	AT2G24570	0.16	0.29	1.01	0.25	WRKY DNA-binding protein 17
*ANAC029*	AT1G69490	−0.94	−0.95	−1.54	−9.57	NAC-like, activated by AP3/PI
*MYB74*	AT4G05100	−9.32	−1.27	−0.27	−0.12	MYB domain protein 74
*MYB59*	AT5G59780	−0.50	−1.64	−1.93	0.19	MYB domain protein 59
*NAC096*	AT5G46590	0.00	−0.96	−1.61	−0.91	NAC domain containing protein 96
*NAC069*	AT4G01550	0.34	0.11	−1.26	0.04	NAC domain containing protein 69
*GPL3*	AT2G36340	−0.23	−0.52	−1.14	−0.09	DNA-binding storekeeper protein related transcriptional regulator
*AtbZIP63*	AT5G28770	−0.03	−0.19	−1.09	−0.38	bZIP transcription factor family protein
-	AT1G60240	−0.36	−0.52	−1.02	0.32	NAC domain transcriptional regulator superfamily protein
*PTAC16*	AT3G46780	−0.38	−0.48	−1.02	−0.08	Plastid transcriptionally active 16
*WRKY49*	AT5G43290	0.23	0.04	−1.00	−0.01	WRKY DNA-binding protein 49

**Table 2:** Differentially expressed genes related to pollen development and function in *Arabidopsis* *thaliana* anthers following ABA treatment.

Gene Name	Gene ID	log_2_ FC				Description	Expressing Localization
		ABA_0.5 h vs. UC_0 h	ABA_2 h vs. UC_0 h	ABA_8 h vs. UC_0 h	ABA_22 h vs. UC_0 h		
*MYB2*	AT2G47190	0.67	1.08	0.20	0.43	MYB domain protein 2	Tapetum
*YUC6*	AT5G25620	0.01	0.44	1.62	0.62	Flavin-binding monooxygenase family protein	Microspores
*P5CS2*	AT3G55610	0.89	1.15	0.79	0.15	Delta 1-pyrroline-5-carboxylate synthase 2	Microspores and pollen
*NAS3*	AT1G09240	0.59	1.02	1.40	−0.73	Nicotianamine synthase 3	Pollen tube
*MYB80*	AT5G56110	−0.71	0.34	0.39	−1.56	MYB domain protein 80	Anther wall tapetum
*QRT2*	AT3G07970	−0.09	0.51	1.32	0.13	Pectin lyase-like superfamily protein	Microspores and anther tapetum
*FST1*	AT5G28470	−0.38	−0.59	−1.24	−0.45	Flavonol sophoroside transporter 1	Anther
*GLP10*	AT3G62020	0.51	1.00	0.89	0.19	Germin-like protein	Pollen exine and pollen tube
*EXL4*	AT5G09440	1.02	1.35	0.52	0.09	Exordium-like 4	Pollen tube
*CSLA07*	AT2G35650	0.19	−0.06	−1.42	−0.12	Cellulose synthase-like protein A07	Pollen tube
*LRX11*	AT4G33970	−0.33	−0.49	−1.11	−0.43	Leucine-rich repeat/extensin 11	Pollen tube
*PMEI1*	AT1G48020	−0.33	−0.45	−1.04	−0.28	Pectin methylesterase inhibitor 1	Pollen tube
*PMEI2*	AT3G17220	0.68	0.56	1.66	1.54	Pectin methylesterase inhibitor 2	Pollen tube
*RABA4D*	AT3G12160	0.33	0.57	1.04	0.37	Rab gtpase homolog A4D	Pollen tube
*CPK14*	AT2G41860	−0.35	−0.54	−1.21	−0.31	Calcium-dependent protein kinase 14	Pollen tube
*PRK4*	AT3G20190	−0.27	−0.43	−1.04	−0.23	Pollen receptor-like kinase 4	Pollen tube

**Table 3:** Differentially expressed lncRNA-target gene pairs involved in stress response in anthers treated with ABA in *Arabidopsis thaliana*.

lncRNA ID	Target DEG ID	Target DEG Name	Description
lncRNA–TF gene pairs			
MSTRG.4503	AT1G69120	*AP1*	K-box region and MADS-box
MSTRG.4503	AT1G68920	*bHLH49*	basic helix-loop-helix (bHLH) DNA-binding superfamily protein
MSTRG.16048	AT4G36710	*ATHAM4/SCL15*	GRAS family transcription factor
MSTRG.4523	AT1G69310	*WRKY57*	WRKY DNA-binding protein 57
MSTRG.4551	AT1G69490	*ANAC029*	NAC-like, activated by AP3/PI
lncRNA–ABA-responsive gene pairs			
MSTRG.3200	AT1G49720	*RCI2A*	abscisic acid-responsive element-binding factor 1
MSTRG.9370	AT3G05880	*ABF1*	dehydration and salt stress and ABA protein family
lncRNA–floral development gene pairs			
MSTRG.4503	AT1G68990	*RPOTM*	male gametophyte defective 3
MSTRG.4503	AT1G69120	*AP1*	K-box region and MADS-box
MSTRG.4551	AT1G69490.	*ANAC029*	NAC-like, activated by AP3/PI

## Data Availability

Raw RNA-seq data from ABA-treated mature anthers of *Arabidopsis* have been deposited in the NCBI Sequence Read Archive (SRA) under Bioproject PRJNA1414170 (https://www.ncbi.nlm.nih.gov/bioproject/PRJNA1414170/, accessed on 26 January 2026).

## Supplementary Materials

The following supporting information can be downloaded at https://www.mdpi.com/article/10.3390/plants15060894/s1, Table S1: Differentially expressed genes in mature anthers of *Arabidopsis thaliana* following ABA treatment; Table S2: Long non-coding RNAs (lncRNAs) identified in mature anthers of *Arabidopsis thaliana* before and after ABA treatment; Table S3: Differentially expressed lncRNAs in mature anthers of *Arabidopsis thaliana* following ABA treatment; Table S4: List of DEL–DEG pairs in mature anthers of *Arabidopsis thaliana* following ABA treatment; Table S5: Clustered expression profiles of DELs obtained by STEM based on the *p*-value significance; Table S6: DELs from significantly clustered expression profiles 1 and 8 obtained by STEM; Table S7: Weighted gene co-expression network analysis (WGCNA) of DELs and DEGs in mature anthers of *Arabidopsis thaliana* following ABA treatment; Table S8: Primers used for qRT-PCR analysis of DEGs and DELs.

## Abbreviations

The following abbreviations are used in this manuscript:

ABA	Abscisic acid
CPC	Coding Potential Calculator
CNCI	Coding–Noncoding Index
DEGs	Differentially Expressed Genes
DELs	Differentially expressed lncRNAs
FPKMs	Fragments Per Kilobase of transcript per Million mapped reads
TF	Transcription factor
GO	Gene Ontology
KEGG	Kyoto Encyclopedia of Genes and Genomes
lncRNAs	Long non-coding RNAs
phasiRNAs	Phase secondary small interfering RNAs
lincRNAs	long intergenic lncRNAs
incRNAs	intronic lncRNAs
PEP carboxylase	Phosphoenolpyruvate carboxylase
RuBP carboxylase/oxygenase	ribulose-1,5-bisphosphate carboxylase/oxygenase
MAPK	Mitogen-activated Protein Kinase
STEM	Short Time-series Expression Miner
WGCNA	Weighted correlation network analysis
ME	module eigengene
